# High resolution transcriptome maps for wild-type and nonsense-mediated decay-defective *Caenorhabditis elegans*

**DOI:** 10.1186/gb-2009-10-9-r101

**Published:** 2009-09-24

**Authors:** Arun K Ramani, Andrew C Nelson, Philipp Kapranov, Ian Bell, Thomas R Gingeras, Andrew G Fraser

**Affiliations:** 1 Donnelly CCBR, College Street, University of Toronto, Toronto, M5S 3E1, Canada; 2 The Wellcome Trust Sanger Institute, Hinxton, Cambridge, CB10 1SA, UK; 3 Department of Physiology, Development and Neuroscience, University of Cambridge, Cambridge, CB2 3DY, UK; 4 Affymetrix, Inc., Central Expressway, Santa Clara, CA 95051, USA; 5 Helicos Biosciences Corporation, Cambridge, MA 02139, USA; 6 Cold Spring Harbor Laboratory, Cold Spring Harbor, New York, NY 11724, USA

## Abstract

**Background:**

While many genome sequences are complete, transcriptomes are less well characterized. We used both genome-scale tiling arrays and massively parallel sequencing to map the *Caenorhabditis elegans *transcriptome across development. We utilized this framework to identify transcriptome changes in animals lacking the nonsense-mediated decay (NMD) pathway.

**Results:**

We find that while the majority of detectable transcripts map to known gene structures, >5% of transcribed regions fall outside current gene annotations. We show that >40% of these are novel exons. Using both technologies to assess isoform complexity, we estimate that >17% of genes change isoform across development. Next we examined how the transcriptome is perturbed in animals lacking NMD. NMD prevents expression of truncated proteins by degrading transcripts containing premature termination codons. We find that approximately 20% of genes produce transcripts that appear to be NMD targets. While most of these arise from splicing errors, NMD targets are enriched for transcripts containing open reading frames upstream of the predicted translational start (uORFs). We identify a relationship between the Kozak consensus surrounding the true start codon and the degree to which uORF-containing transcripts are targeted by NMD and speculate that translational efficiency may be coupled to transcript turnover via the NMD pathway for some transcripts.

**Conclusions:**

We generated a high-resolution transcriptome map for *C. elegans *and used it to identify endogenous targets of NMD. We find that these transcripts arise principally through splicing errors, strengthening the prevailing view that splicing and NMD are highly interlinked processes.

## Abstract 2

The high-resolution transcriptome of wild-type and nonsense-mediated decay (NMD) defective C. elegans during development reveals insights into the NMD pathway and it’s role in development.

## Background

Identifying genes whose mRNA expression is perturbed in a mutant can yield great insight into a wide range of biological problems. For example, comparing gene expression in wild-type organisms with that seen in mutants can be used to identify the targets of transcription factors or signaling pathways [1], to organize genes into modules [2-7], and to order genes in pathways [8,9]. Recently, genome-scale tiling arrays and massively parallel sequence analysis of transcriptomes have emerged as powerful new tools for transcriptome analysis [10-14]. Both rely on the availability of high quality genome sequence, and both offer the promise of transcriptome analysis at unprecedented depth and efficiency.

Each technology has different strengths. In the case of tiling arrays, the entire transcriptome can be queried at the same depth in a single hybridization, making it a very cost-effective way to achieve excellent coverage. However, the resolution with which any transcript can be mapped is limited by the resolution of the array (which for most complex genomes is not at single base-pair resolution) and, furthermore, while one can rapidly identify the regions of the genome that correspond to mature transcripts, the arrays contain no implicit information about how these are connected. Deep sequencing of the transcriptome on the other hand generates data at single-base resolution. While the sequence reads from all current technologies are short (typically 35 to 70 bp), it is possible to assemble these into longer contiguous reads and to link these contigs together. However, since the range of gene expression extends over many orders of magnitude, achieving good coverage for a complex transcriptome is still costly, and assembly of the data is still computationally intensive.

Since tiling arrays and sequencing have complementary benefits for transcriptome analysis, we decided to use both technologies to examine the *Caenorhabditis elegans *transcriptome across a series of developmental stages. The *C. elegans *genome is completely sequenced and while it contains a similar number of genes as the human genome, it is much more compact - around 27% [15] of the worm genome is coding compared with 1.5% [16] in humans. Genome annotation is generally of high quality in the worm; the genome is relatively small (approximately 100 Mb compared with approximately 3 Gb in human) and unrepetitive, making both tiling- and sequence-based approaches comparatively straightforward in the worm. Both technologies allow examination not only of levels of gene expression but also of splice changes across development; they also allow identification of novel transcripts that do not lie in annotated gene structures. They thus provide an unbiased and rich view of the changing transcriptome across development and our immediate goal was to map the wild-type transcriptome at good coverage and resolution and, thus, to provide a framework to analyze perturbations of the transcriptome in mutants.

In addition to mapping the wild-type transcriptome across several developmental stages, we wished to assess the usefulness of these data for examining how the transcriptome is perturbed in mutant animals. To this end, we used both tiling arrays and sequencing to examine the transcriptome of worms defective for nonsense-mediated decay (NMD), identified in animals by Hodgkin *et al. *[17] and reviewed in [18,19]. The central cellular role of the NMD pathway is to prevent the expression of prematurely truncated proteins, which are likely to have deleterious consequences. The NMD pathway recognizes transcripts containing premature termination codons (PTCs) and targets them for degradation, thus eliminating them from the cell [20]. The role of the NMD pathway in eliminating PTC-containing transcripts is highly conserved and indeed many of the components are shared from yeast to human (see [21,22] for reviews), including the core components SMG-2, SMG-3 and SMG-4 (Upf1-3 in *Saccharomyces cerevisiae*).

The PTC-containing transcripts that are targets for NMD recognition and degradation arise from three principal sources [21,23-27]. The first occurs from transcripts deriving from genes containing nonsense mutations, whether inherited or somatic. However, nonsense mutations play a clear role in many human genetic diseases, and in several of these, NMD has been shown to affect the severity of the disease phenotype and, thus, this class of target, though rare, has key medical importance [28]. The second class comprises transcripts that contain PTCs that arise during alternative splicing - either retention of introns or errors in splice site selection [29-32]. Finally, transcripts can be targeted by NMD despite having no PTCs in the principal open reading frame (ORF); instead, these transcripts contain a short ORF upstream of the true start ATG, known as an upstream ORF (uORF). The stop codon of this uORF is recognized as a premature stop codon and the transcript is thus recognized as an NMD target. Recently, genome-scale studies using standard expression microarrays have identified endogenous transcripts that are targets of NMD in yeast [33,34], *Drosophila *[35,36], and humans [37-39]. In all three organisms examined, approximately 10% of genes give rise to a transcript that is targeted for degradation via the NMD pathway, a surprisingly large number [40].

Much is thus known already about NMD: the molecular components of the NMD pathway are well-characterized, many of the molecular features that cause a specific transcript to be degraded via the NMD pathway are known, and many endogenous transcripts are found to be affected by NMD. However, other than in yeast [32] no genome-scale studies have examined the effect of NMD on wild-type transcriptomes with the resolution that either tiling arrays or transcriptome sequencing can provide. We thus set out to compare the transcriptomes of wild-type animals with that of worms that are defective for NMD using both tiling arrays and deep sequencing to determine whether the increased resolution of such analyses can provide new insight into the effect of NMD on the transcriptome of normal developing worms.

## Results and discussion

### Outline of approach and overview of data

Both genome-scale tiling arrays and deep sequencing approaches were used generate a high resolution, high coverage 'reference transcriptome' for *C. elegans *to use as a tool to guide analysis of perturbed transcriptomes such as those of mutant animals. At the time of initiating these studies, there was a great difference in the cost to analyze any specific RNA sample by tiling arrays or by deep sequencing, and we thus chose to use tiling arrays as our primary method to map the transcriptome across multiple developmental stages, and deep sequencing to validate the tiling data and to refine the resolution of the transcript mapping at a more limited number of developmental stages. Combining these data in this way combines the cost-effectiveness of tiling with the higher resolution of sequencing to generate a high quality transcriptome map.

For our tiling analysis, we purified total RNA from wild-type N2 animals at four different stages of the *C. elegans *life-cycle (larval stages L3 and L4, young adults, and gravid adults). For each developmental stage, RNA samples were prepared in triplicates and hybridized individually to genome-scale tiling arrays - these have a 35 bp resolution and allow an unbiased view of the majority (70%) of the genome. We initially examined these data to assess coverage and to compare data quality between tiling and sequencing. At any single developmental stage, we detect expression of around a third of all predicted genes on tiling arrays (see Materials and methods; Table S1 in Additional data file 1); across all examined stages, we detect approximately 50% (9,515 out of 19,169 annotated genes in WS150 release of Wormbase [41]) of genes. This is comparable to the detection sensitivity of conventional microarrays. We find that approximately 95% of transcribed features (so-called 'transfrags', the individual contiguous regions of the genome that are transcribed; see Materials and methods and [11,13] for definition) map to currently predicted transcripts (Table S2 in Additional data file 1), a far higher proportion than that observed in either *Drosophila *[42] or human [10]. We note that while the proportion of novel transfrags is far lower in the worm, this is in keeping with previous results [12] and is broadly as expected for the worm genome given the far higher proportion of predicted coding sequence relative to that found in many other animal genomes. In addition, this low proportion of novel transcribed regions identified as transcribed indicates that genome annotation and gene prediction in the worm is of generally excellent quality.

**Figure 1:**
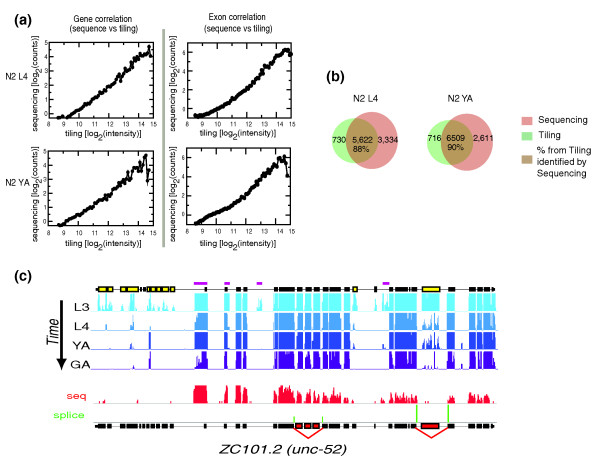
Tiling array data and sequence-based data give similar views of the transcriptome. **(a) **Gene intensities (left) and exon intensities (right) from the tiling data were binned at 0.1 increments of gene intensity (log_2 _scale) and compared with the intensities deriving from sequence data; there is a strong correlation (R^2 ^= 0.95) between gene intensities derived from both technologies. YA, young adult.**(b) **Approximately 90% of the genes expressed based on tiling are also expressed in the sequence data in both stages sequenced. **(c) **Sample screenshot from Affymetrix Integrated Genome Browser illustrating how tiling array data and sequence data correspond to predicted gene structures. Tiling array data from four developmental stages (L3 and L4 larvae, YA and gravid adults (GA)) are shown in shades of blue. The predicted exons of *unc-52 (ZC101.2) *are shown at the top of the plot. Exons that are differentially spliced across development based on tiling data are shown in yellow. Regions corresponding to transfrags that do not overlap predicted exon structures are highlighted with purple bars. Sequence data for a single developmental stage (YA) is shown in red at the bottom of the figure; note that the regions identified as transcribed by sequencing correspond closely to those identified by tiling. Non-adjacent exon boundaries spanned by sequence reads are shown as green bars and the exons removed by the alternative splice shown in red below; the height of the green bar corresponds to the frequency with which the alternative splice events were detected.

To validate our tiling data, we used Illumina sequencing to directly sequence the transcriptome of two developmental stages (L4 and young adults) along with mixed stage worms and compared these sequence data to the tiling data. We generated approximately 225 million individual reads, of which approximately 217 million (85%) could be uniquely assigned to the genome using Mapping and Assembly with Qualities (MAQ; Table S3 in Additional data file 1) [43]. Of these uniquely mapped reads, approximately 94% map entirely within known transcripts (approximately 85% of all reads map entirely within known exons and approximately 9% span exon-exon junctions), a number that corresponds closely to that seen by tiling (95% of transcribed regions map to known transcripts by tiling). We compared gene intensities deriving from both tiling and sequence data and find very tight correspondence between these measurements (Figure 1a), suggesting that both methods give accurate estimates of levels of mRNA expression. Finally, we compared the sets of genes whose expression is detected by tiling and by sequence analysis and find that approximately 90% of genes that have detectable expression by tiling can also be detected by sequence (Figure 1b). We thus show that the two technologies provide accurate and complementary surveys of the transcriptome, allowing direct comparison with the transcriptomes of mutant animals.

### Novel transcribed regions of the *C. elegans *genome

**Figure 2:**
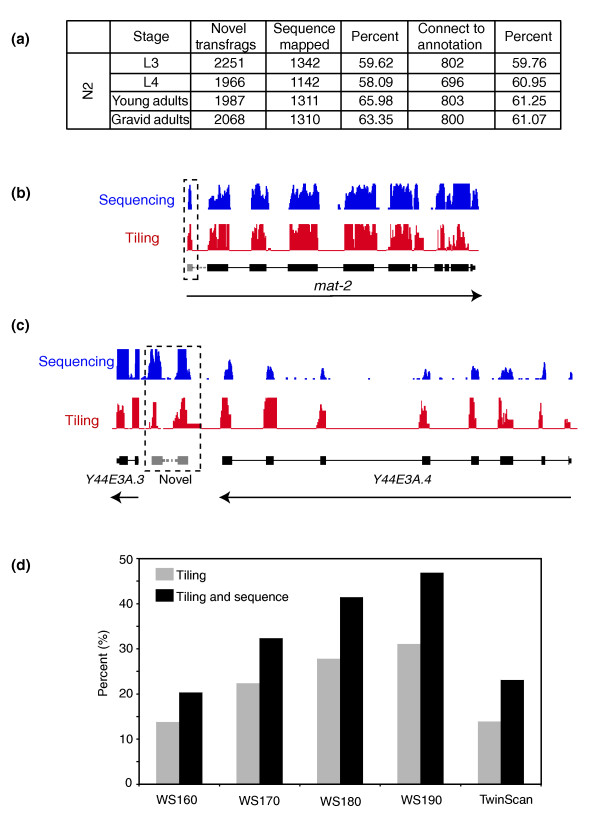
Novel transfrag annotation. **(a) **Using stage specific sequence data we were able to show that approximately 60% of the novel transfrags have sequenced reads mapping to them. We also show that 60% of these transfrags with sequence information can be connected to known gene annotation using paired-ended sequence reads, where one read of the pair is anchored on the transfrag while the other overlaps a gene annotation. Examples of novel regions identified from our analysis, show **(b) **a new 5' exon and **(c) **a novel transcript. **(d) **Transfrags identified as novel in our tiling data based on WS150 of the genome annotation were compared against WS160, WS170, WS180 and WS190 models. We see that >30% of the transfrags that were novel based on WS150 are predicted to be exonic in later annotations (grey bars). Almost 50% of novel transfrags that also have sequence reads overlapping them are predicted to be exonic in later annotations (black bars). We can show annotation overlap for a further 15% (tiling alone - gray) or 25% (tiling with sequence data - black) when we compare the transfrags to TwinScan models.

As described above, we find that approximately 95% of the transcribed regions detected by either tiling array or sequence map to predicted transcripts (Additional data file 2) - a representative region of the genome is shown in Figure 1c. We next examined whether both technologies detected the same novel transcribed regions and whether these novel regions are likely to represent entirely new stand-alone transcripts (that is, from potentially new genes) or rather are novel exons of previously annotated genes. We first identified all novel transfrags identified on tiling arrays at any developmental stage by comparing the tiling data to WS150 gene models and asked what proportion of these can be confirmed by sequence reads (Figure 2a; Table S4 in Additional data file 1). Of the novel transfrags found by tiling, approximately 60% can be detected by sequence - since both technologies are very different, these confirmed novel transfrags are likely to be real. Note that while tiling arrays were used to analyze total RNA, only poly-adenylated transcripts were sequenced to avoid redundant reads of rRNA, and this is thus a lower estimate of true novel transcripts identified by tiling. We note that two other studies have appeared that also used sequencing to analyze the *C. elegans *transcriptome and we thus compared our data with that produced by Hillier *et al. *[44], who also used deep sequencing to examine the transcriptome at several developmental stages (rather than Shin *et al. *[45] who examined only L1, a stage we did not look at). We find that the overlap is very significant at the gene level (Table S5 in Additional data file 1) and at the level of transfrags (Table S6 in Additional data file 1), confirming the accuracy of all datasets.

Novel transfrags can either arise from entirely new transcripts that have not been predicted or they could alternatively be novel exons or previously predicted genes. In the latter case, it should be possible to connect these novel transfrags to known gene annotations. To examine this, we used Illumina paired end sequencing on poly-A+ RNA derived from mixed stage populations of worms. We identified reads mapping to novel transfrags and asked whether the paired sequence read mapped to a known gene structure. In approximately 60% of cases (Figure 2a), we could unambiguously connect a novel transfrag confirmed by both sequence and tiling to a previous predicted transcript, suggesting that these are novel exons (Figure 2b, c). Of such novel exons, 65% (20% 5' and 45% 3') are either 5' or 3' to the coding region of the gene, consistent with a view that terminal exons are more variable; therefore, predicting transcript ends is considerably harder and more error prone than predicting internal coding exons.

Finally, to further investigate the novel transfrags identified by tiling, we compared our tiling data to multiple other gene models in *C. elegans*. First, we examined the proportion of our novel transfrags (based on gene models in version WS150 of Wormbase) that were still novel in later sets of gene models (WS160, WS170, WS180, and WS190) (Figure 2d). We find that approximately 30% (670 of 2,229) of transfrags that were outside gene models in WS150 have since been incorporated in newer gene models; 90% of those that are novel exons are now confirmed in gene models. Of the remaining (approximately 70%; 1,530 of 2,229), we note that many map to alternative gene models outside the canonical *C. elegans *gene models - for example, approximately 15% (204 of 1,530) overlap with Twinscan models. We believe that many of these novel transfrags are likely to represent errors in standard gene models, since other gene models predict many of them relatively well. Thus, our data, like previous work [44,45], may contribute to refining *de novo *gene models.

In total, then, we identified 10,073 (the unique non-overlapping set from the four stages) novel transcribed regions relative to gene models in WS150 using tiling arrays. Most of these (Table S4 in Additional data file 1) could be confirmed by sequence and of those identified by both technologies, approximately 30% appear to be novel exons of previously annotated transcripts.

### Alternative splicing detection using tiling arrays and transcriptome sequencing

Tiling arrays can be used to measure gene expression at the level of mRNA. However, unlike conventional expression arrays, tiling arrays can also be used to examine the expression of individual exons and their relative inclusion into transcripts deriving from any gene. Changes in the relative inclusion of an exon across development indicate changes in splicing and we thus investigated the extent to which we could identify splice changes across *C. elegans *development using our tiling data. For each exon, we computed its normalized intensity at each developmental stage based on tiling data. The normalized intensity (NI) of any exon is the expression level of the exon relative to the expression level of the gene that includes it. An NI of approximately 1 for an exon indicates that essentially all transcripts deriving from that gene include that exon; an NI of approximately 0 indicates that this exon is skipped from almost all transcripts deriving from that gene. We note that just as gene expression levels measured by tiling and sequence correlate very highly, this is also the case for levels of expression of each individual exon (Figure 1a).

**Figure 3:**
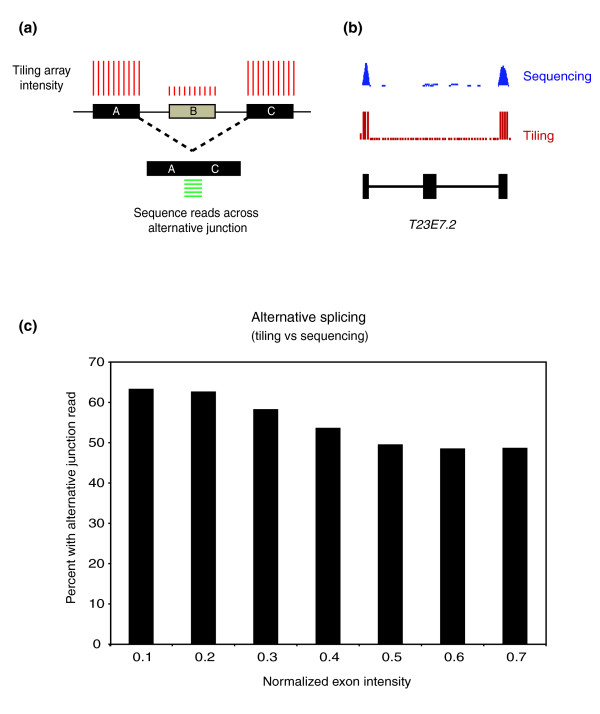
Splicing changes. Normalized exon intensities (see Materials and methods) were calculated for all exons. **(a) **The set of cassette exons was determined using our tiling data as exons with an NI <0.8 (exon B). Cassette exons may also be indicated by the presence of sequence reads spanning the boundaries of the flanking exons (exons A and C). **(b) **An example of a typical cassette exon. **(c) **Exons were binned based on their NI (x-axis) and the percentage of these cassette exons that have sequence reads spanning the exonA-exonC junction were identified. At a NI <0.2 nearly 65% of the cassette events show an alternative exon read while 50% of all exons with NI <0.5 can be shown to have an alternative junction spanning read.

Prior to examining how the NI of each exon changes across development, we compared exon inclusion as estimated by NI from tiling with direct measurements of splicing from our sequence data. We identified sequence reads that span exon-exon junctions - we searched for these both between adjacent exons and between non-adjacent exons (see Materials and methods). Note that reads spanning exon-exon junctions are far more rare than those mapping internally to exons since the effective target is smaller and achieving high coverage of exon junctions thus requires substantially more sequence depth than that required simply to detect gene expression; furthermore, identification of such exon spanning reads is highly sensitive to correct exon junction predictions. To examine the extent to which NI measured from tiling data gives a verifiable measure of splice variation, we identified all exon triplets that appear 'cassette-like' from our tiling data (see Figure 3a for schematic and Figure 3b for an example) - that is, triplets where exons A and C have NI of approximately 1 and the middle exon B has an NI of <1. In such cases, we would infer that A and C are included in all transcripts and that B is included at a lower rate due to exon skipping as measured by its NI. This is the simplest test example. To test whether the alternative splicing that we infer from tiling NI data is accurate, we examined all such triplets to determine how often we could identify reads that provide direct evidence for skipping of exon B, identified by detecting sequence reads spanning the junctions of A-C. These data are shown in Figure 3c and show that we can identify sequence reads, confirming at least partial exon skipping for over 65% of exons with an NI <0.2. This drops to approximately 50% for exons with an NI <0.5; 688 exons have an NI <0.5, corresponding to 635 genes, and, based on these data, at least 50% of these alternative splicing events can be confirmed by sequence. We note that the ability to confirm alternative splicing events based on tiling arrays by sequence reads is highly dependent on read depth and on the NI of the exon in question and this is thus likely to be a considerable underestimate of the true quality of the tiling-based splicing analysis.

Having established that normalized exon intensity can identify alternatively spliced exons with reasonable accuracy, we searched for exons whose NI changes between any two developmental stages. A significant change in NI for an exon between two stages indicates a shift in isoform levels for the gene in question. Note that we cannot infer the isoforms themselves, but only identify that they have changed based on changing NI. We find that approximately 5% (459 of 9,515) of expressed genes contain at least one exon whose NI changes by over 2-fold between any two developmental stages - that is, at least approximately 5% of genes change their relative isoform levels across development. While approximately 18% (based on WS150 release of the genome annotation) of worm genes are currently annotated to have multiple isoforms, this is the first genome-scale analysis of how isoforms change across development, although the identification of 5% of genes whose isoform patterns change across development is highly likely to be an underestimate.

### Identification of endogenous targets of nonsense-mediated decay

As described above, we have combined tiling arrays and deep sequencing to construct a high-resolution reference transcriptome for wild-type animals across several developmental stages. We wished to use this to examine how the transcriptome is perturbed in mutant animals. Specifically, we were curious to see whether the high resolution of our transcriptome map could yield insights that would not have been evident using standard gene expression microarrays. As a test case, we chose to examine the transcriptome of worms defective for NMD. This was first identified in animals by Hodgkin *et al. *[17] and is a highly conserved cellular program evolved to prevent the expression of prematurely truncated proteins (reviewed in Chang *et al. *[18]) The NMD pathway recognizes transcripts containing PTCs and targets them for degradation - thus, transcripts that are targets for NMD should have elevated expression in cells that have no functional NMD pathway. This has been used to identify endogenous transcripts that are NMD targets in *S. cerevisiae*, *Drosophila *and human cells [34,36-39,46]. We mapped the transcriptome of worms that are defective for NMD using a combination of tiling arrays and sequencing (exactly as was done for the wild-type transcriptome above), and compare it to our wild-type reference transcriptome (Additional data file 3). This should allow us first to identify the endogenous targets of NMD in *C. elegans*, which has never been done at the genome-scale; second, to examine the features of transcripts are endogenous targets of NMD in *C. elegans *to see whether this is similar to that found in other organisms; and finally to determine whether we can gain novel insights from our high resolution map that are not seen simply by examining overall gene expression.

We examined the transcriptome of *smg-1(r861) *mutant worms - *smg-1 *encodes a central kinase in the NMD pathway, is highly conserved in eukaryotes, and is absolutely required for NMD in *C. elegans *[17]. We purified total RNA from *smg-1(r861) *mutant animals at the same four developmental stages (L3, L4, young adults, and gravid adults) examined in the wild-type animals and hybridized these in triplicate to genome-wide tiling arrays. We computed gene intensities as for the wild-type transcriptome and could thus identify genes whose expression levels are perturbed in the *smg-1(r861) *mutants. We also used Illumina sequencing to examine the *smg-1(r861) *transcriptome at two developmental stages to check our tiling data. We find that just as gene expression levels and exon expression levels were highly similar between tiling and sequencing in the wild-type transcriptome, they are highly correlated for the *smg-1(r861) *data (Additional data file 3).

Looking across all developmental stages, approximately 17% (1,645 of 9,515) of all detectable genes differ in expression level by at least 1.5-fold between wild-type and *smg-1(r861) *worms and in the great majority of cases (approximately 75% overall), transcript levels are higher in the *smg-1(r861) *mutant, consistent with these being NMD targets. To confirm that these are not somehow specific to the *smg-1(r861) *strain, we also examined one time point (L4 larvae) in animals mutant for SMG-5 [47], a key phosphatase in the NMD pathway, and find that the great majority (318 out of 437 genes in this stage; approximately 73%) of genes whose expression differs between wild-type and *smg-1(r861) *animals also differs between wild-type and *smg-5 (r860) *animals [47], confirming the majority of these differences are indeed the result of loss of NMD and are not somehow specific to *smg-1(r861)*. We thus estimate that at least 10% of genes produce a transcript that is elevated in an NMD mutant animal; this is likely to be an underestimate. This is a very similar proportion to that seen in yeast, fly, and human and suggests that while genome complexity and transcriptional regulation is very different in these organisms, the proportion of genes whose expression is affected by NMD is very similar. We next sought to examine the features of the transcripts that have elevated expression in the *smg-1(r861) *mutants.

### Features of NMD-regulated transcripts in *C. elegans *identified from gene models

We have identified many genes whose expression is increased in animals that have no functional NMD pathway. We note that identifying a higher level of expression of any gene in a NMD mutant does not mean that every transcript deriving from that gene is an NMD target, nor can we be sure that the effect is direct. However, what is clear is that all true endogenous NMD targets (ENTs) will have increased expression in mutant animals where NMD is completely lost. We therefore reasoned that transcript features associated with true NMD targets will be enriched in the sets of transcripts that are more highly expressed in the *smg-1(r861) *mutants even if some expression changes are the result of downstream effects.

**Figure 4:**
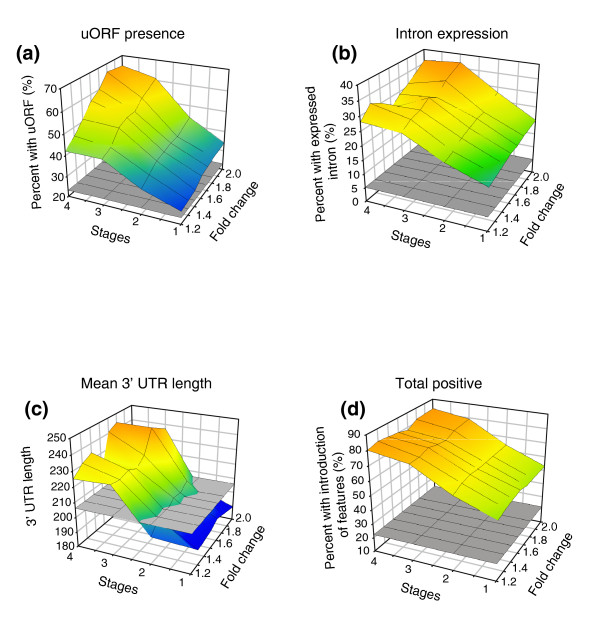
Features predisposing transcripts to NMD. Indicated are the percentage of genes exhibiting the measured feature (y-axis), the number of stages at which this is observed (x-axis) and the fold increase in gene intensity in *smg-1(r861) *over N2 (z-axis, log_2 _scale). In each case the average background occurrence of the feature is indicated by the grey square. The measured features are: **(a) **percentage of genes with a uORF; **(b) **percentage of genes with expressed introns; **(c) **average 3' UTR length; **(d) **the total percentage occurrence of the above three NMD features for the set of over-expressed genes. The plots show a clear positive correlation between the feature examined and the increased effect of NMD on the transcripts.

**Figure 5:**
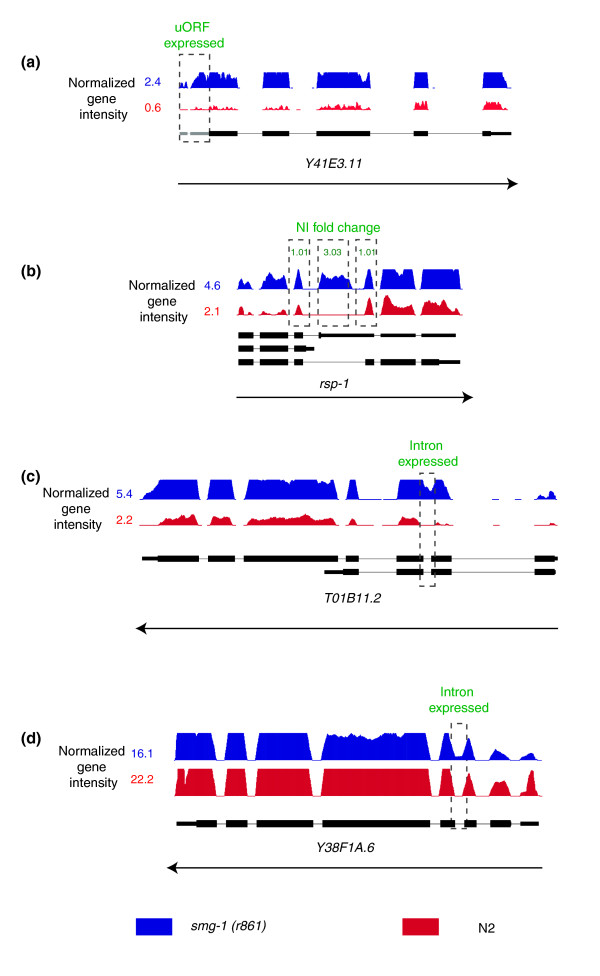
Examples of NMD features. **(a) **Example of a gene upregulated in *smg-1(r861) *and showing transcript expression from the upstream ORF. **(b) **Exon4 of *rsp-1 *is alternatively spliced in *smg-1(r861) *animals, observed as a change in the NI of the exon between the two stages. **(c) **The retention of an intron expressed at very high levels in the mutant, which also has a higher gene intensity. **(d) **A retained intron, where the gene intensity remains similar between the mutant and wild type (that is, a gene for which only a small proportion of transcripts retain the intron).

We examined the predicted transcript structures of all 1,645 genes showing higher expression in the *smg-1(r861) *animals to identify enriched features. As shown in Figure 4, we find strong enrichment for two features: the presence of an uORF in the 5' untranslated region (UTR) upstream of the predicted start ATG (Figure 4a; Additional data file 4) and 3' UTR length (Figure 4c; Additional data file 4). The greater the effect of loss of NMD on the transcript level, and the greater the number of developmental stages for which we see this change, the stronger the enrichment appears to be. This is consistent with previous analyses in other organisms and confirms that we can identify transcript features that appear to be responsible for targeting endogenous transcripts by NMD by searching for features that are enriched in transcripts that are more highly expressed in the *smg-1(r861) *animals [24-27,36,48]. To identify this enrichment of uORFs and longer 3' UTRs in ENTs, we relied exclusively on gene models. We next wished to use our tiling and sequence data to look at other transcript features. In particular, many PTCs are introduced by splicing errors [29,31,32,40,49,50], including intron retention and the incorrect retention or skipping of exons, and we examined these individually and show examples in Figure 5.

### Intron retention in NMD-regulated transcripts in *C. elegans*

**Figure 6:**
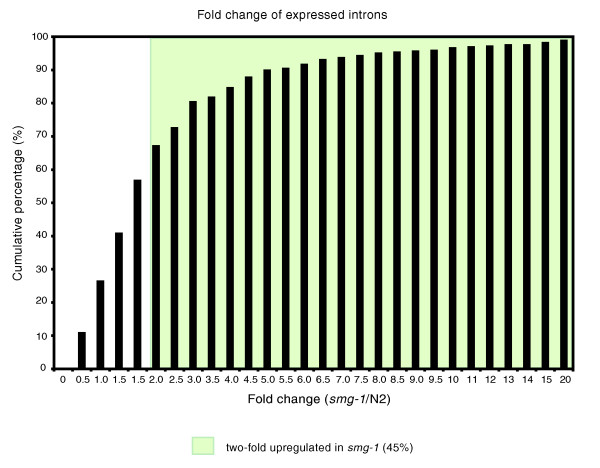
Intron expression is upregulated in NMD mutants. Of the introns expressed in both N2 and *smg-1(r861)*, 1,642 (45%) introns are ≥2-fold upregulated in *smg-1(r861)*. Nearly 75% of the expressed introns have higher expression (>1 in the x-axis) in *smg-1(r861) *compared to wild type.

We computed intensities from all annotated introns using identical methods to those for computing exon intensities (see Materials and methods). If there is detectable expression of any individual intron, we conclude that it is being at least partly retained in transcripts deriving from that gene; in the vast majority of cases (>99%), such retention introduces a PTC. We find that genes that have higher expression levels in *smg-1(r861) *mutant animals are highly enriched for the presence of a retained intron (Figure 4b; Additional data file 4), confirming that intron retention is a major cause of PTCs and, hence, a feature of ENTs in the worm. This analysis to identify intron-containing ENTs is relatively insensitive, however; if only a very small fraction of transcripts deriving from any one gene retain an intron, this will result in only a very small difference in gene expression between wild-type worms and *smg-1(r861) *mutants and we would thus not detect this. In addition to asking 'of the genes with increased expression in *smg-1(r861) *mutants, what proportion have retained introns?', we can ask 'are there introns whose expression levels differ between wild-type and *smg-1(r861) *mutants?' This is a far more sensitive analysis since it can pick up introns that are retained in only very low fractions of transcripts coming from any gene but whose retention causes NMD to target those rare transcripts. We find 1,640 introns that have at least 2-fold increased expression in *smg-1(r861) *mutants (Figure 6); these correspond to transcripts from 1,274 genes. In almost all cases, while the intron-containing transcripts are NMD targets, they are expressed at very low levels compared to the overall gene intensity and the great majority of genes with such introns thus have essentially identical expression in wild-type and *smg-1(r861) *animals.

We conclude that precise intron excision is relatively efficient in the worm since the great majority of introns are correctly excised and thus undetectable. However, at least 7% (1,274 of 20,000) of genes produce transcripts in which an intron has failed to be excised and that are ultimately degraded by NMD in wild-type animals. Intriguingly, we find that the retained introns are not a random set, but instead share certain features. Most particularly, we notice a decrease in usage of the canonical TTTCAG splice site consensus at the 3' end of these introns and a corresponding increase in the usage of less common splice sites. While nearly 75% of annotated introns contain the canonical TTNCAG, only 55% of the expressed introns have these 3' splice sites (*P*-value = 0.004) [51]. We thus suggest that the number of errors associated with excision of introns flanked by splice sites that closely match the consensus sequences is far lower than for introns that have more divergent splice sites. This is similar to the relationship between diminished 5' splice donor and branch point consensus and intron retention leading to NMD as observed in yeast [32].

### Splicing changes in NMD-regulated transcripts in *C. elegans*

It is becoming increasingly apparent that splicing and NMD are two highly linked processes, with almost entire families of splicing factors being alternative splicing-dependent NMD targets themselves [29,39,40,52]. We examined differences in splicing between wild-type and *smg-1(r861) *animals at the level of exon intensity. As described above, we computed NIs for all cassette exons in each of the developmental stages in the *smg-1(r861) *animals and compared these NIs to those of each exon in wild-type animals. A difference in NI between wild-type and *smg-1(r861) *animals for any exon indicates a difference in overall levels of inclusion of that exon in the entire set of transcripts deriving from that gene in worms lacking NMD; this may be a direct or indirect effect of loss of NMD. In the case of direct effects, the exact same set of transcripts is synthesized in wild-type and *smg-1(r861) *animals; however, some splice variants contain PTCs and are thus degraded by NMD in wild-type worms. The difference in NI for an exon between wild-type and *smg-1(r861) *is thus due to the failure to degrade these PTC-containing isoforms in *smg-1(r861) *animals. Retention of these PTC-containing isoforms in *smg-1(r861) *animals will affect overall gene intensity; hence, in the case of splice changes that are the direct effects of NMD, not only will the NI of any exon be perturbed in the *smg-1(r861) *mutant, but the overall gene intensity will also be affected. Alternatively, a change in NI for an exon between wild-type and *smg-1(r861) *might be completely indirect, some downstream consequence of loss of NMD. In such cases, the difference in NI for an exon is due to a difference in the isoforms synthesized between wild-type and *smg-1(r861) *mutants. In these indirect cases, the NI difference reflects a difference in splicing between wild-type and *smg-1(r861) *mutants rather than a difference in transcript turnover/retention, and there will thus be no accompanying difference in gene intensity between wild-type and *smg-1(r861) *mutants. We thus distinguish direct from indirect effects of NMD on splicing patterns by assessing whether there is a gene intensity change accompanying any splice change that we see; if there is a concomitant change in gene expression, we deduce that the splice change is a direct effect of NMD.

We find that 485 genes have an exon whose NI differs between wild-type and *smg-1(r861) *mutants by 2-fold or more. In 350 of these 485 genes (72%) we find that the varying exon has an NI of 0.5 or more in the *smg-1(r861) *mutant; that is, in animals that have lost NMD, that exon is present in at least 50% of the transcripts deriving from that gene. However, only a minority of these genes (approximately 22%) show any difference in expression level in the NMD mutant animals. This is surprising - if these splice changes were the result of retaining transcripts with PTCs, we would expect a substantial difference in overall gene expression levels in the mutant. This suggests that many of the NI changes seen are likely to be indirect effects of loss of NMD (since transcripts are not NMD targets with or without the varying exon) and may be indicative of a more general perturbation in splice site selection in the NMD mutants. One possibility to explain indirect effects on splicing in NMD mutants is that NMD is affecting expression of splice factors themselves and there is previous evidence to suggest this may be true. One particular class of genes whose expression is affected by NMD comprises the *rsp *genes that encode the SR family of splice factors [30,40,50]. Indeed, when we examine our tiling data we see that seven of the eight *C. elegans rsp *genes give rise to NMD-targeted transcripts that have retained introns and we confirmed all these events by RT-PCR (Additional data file 5). We also find that the SR and hnRNP families of splice factors are over-expressed in the *smg-1(r861) *mutants based on both our tiling and sequence data (Table S7 in Additional data file 1), and wanted to extend this analysis to other splice factors to see if this is generally true.

We examined a manually curated list of all well-annotated splice factors (Table S8 in Additional data file 1) to see if NMD has an effect on expression of other splice factors and find that while only 13% (2,631 of 20,000) of all genes have an exon with an NI differing by 1.5-fold or more between wild-type and *smg-1(r861) *worms, approximately 33% (44 of 132; *P*-value < 0.0001) of splice factors have an exon with an NI differing by 1.5-fold. This is a strong enrichment and accords with previous findings [29,30,39,40,50,53]. Crucially, the great majority of these differences in NI in splice factors in *smg-1(r861) *worms appear to be direct consequences of the loss of NMD - while only 30% of genes with strong differences (3-fold or higher) in NI between *smg-1(r861) *and wild-type show any difference in expression level in the NMD mutant animals, over 90% of splice factors with similar NI differences (3-fold) show increased expression levels (1.5-fold or higher) in the *smg-1(r861) *worms. We thus propose that the majority of splice differences between wild-type worms and worms defective for NMD are indirect; however, in the case of splice factors themselves, most splice differences are highly likely to be direct, the immediate result of expression of PTC-containing isoforms of these genes.

### Translational initiation efficiency at the true start ATG affects NMD targeting of transcripts

**Figure 7:**
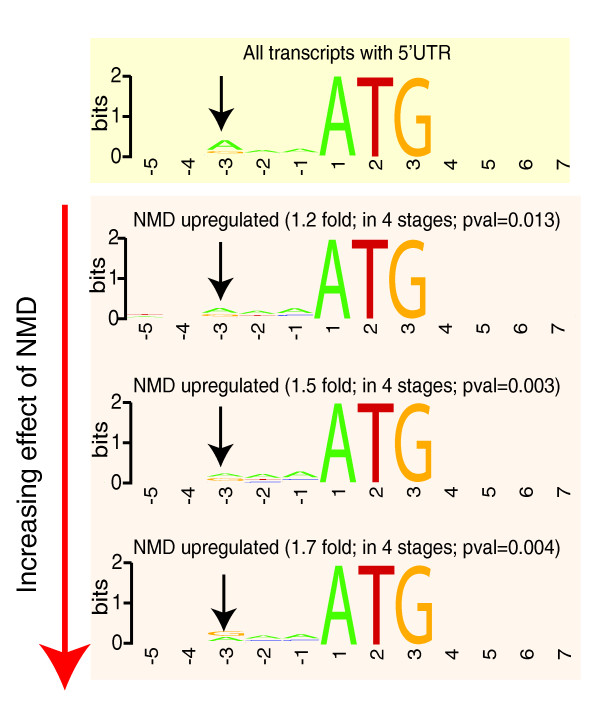
Relationship between consensus Kozak sequence at the true start ATG for genes affected by NMD compared with all genes. In each panel the x-axis corresponds to the sequence considered while the y-axis refers to the percent occurrence at each nucleotide position expressed as 'bits' using Weblogo [70]. The top panel (in yellow) shows the consensus among all transcripts with an annotated 5' UTR and reveals the importance of an adenine in the -3 position - this is the classic Kozak consensus. This occurrence of adenine at the -3 position decreases significantly with increased NMD regulation as is shown in the bottom panel (red). The significance of change in enrichment of the adenine at -3 between NMD regulated and all genes was determined by chi-squared test. Pval, *P*-value.

In the preceding sections, we described the identification of transcript features that are enriched in genes that have increased expression in animals that have no functional NMD pathway. One of the strongest features associated with NMD-regulated transcripts in the worm is the presence of an uORF (see above). Intriguingly, however, while ENTs are enriched for the presence of uORFs, many transcripts that have uORFs are not affected at all by NMD - how does the organism discriminate between these? An obvious explanation for this might be that the extent to which NMD targets an uORF-containing transcript is strongly linked to the efficiency with which the uORF is translated. In this model, in the cases where the uORF is translated efficiently, the NMD effect is strong; in transcripts where the uORF is not translated efficiently - and thus is not 'seen' by the cell - NMD has little effect. One key determinant in the rate of translational utilization of any start ATG is the sequence surrounding the ATG; the best known of these is the Kozak consensus. We thus examined the sequences surrounding the start ATG of the uORF for the set of transcripts that are affected by NMD and those that are not (see Materials and methods) to detect any differences in sequence composition. We find no difference between these two sets of genes. Intriguingly, however, when we examined the sequences surrounding the predicted true start ATG for both ENTs and transcripts unaffected by NMD, we found a clear difference. While 60% of transcripts with annotated 5' UTRs have an adenine at the -3 position (consistent with a Kozak consensus [54]), in the case of the NMD-regulated genes the percentages of adenines at this position are 53%, 47% and 43% for genes with 1.2-fold, 1.5-fold and 1.7-fold higher expression in *smg-1*(r861), respectively (Figure 7). Thus, a weaker Kozak consensus at the true ATG correlates well with a higher effect of NMD on transcript regulation. This fits with a scanning model for ATG selection whereby the start ATG is identified following a pioneering round of translation - if the true ATG is in a strong Kozak consensus, the uORF ATG may not be subsequently used and the transcript may thus evade NMD. We note that this effect is slight, and is poorly predictive - many uORF-containing transcripts that evade NMD do not have a strong Kozak at the true start ATG, and *vice versa*. Thus, this slight trend cannot explain the bulk of the variation in NMD targeting for all examples of uORF-containing transcripts but it does suggest a highly speculative model where a Kozak consensus at the true ATG may influence both translational efficiency and transcript abundance.

## Conclusions

Both genome-scale tiling arrays and new generation sequencing technologies can be used to examine the transcriptome at great depth and coverage. We used both technologies to make a 'reference' transcriptome for *C. elegans*, examining several different developmental stages of wild-type worms using high resolution tiling arrays and validating the data using deep sequencing. Our principal aim was not to make an exhaustive and complete map of the *C. elegans *transcriptome, but rather to generate a high-resolution scaffold that can be used to identify subtle perturbations of this transcriptome in mutant animals. Nonetheless, mapping the wild-type transcriptome itself identified a number of interesting findings and novel features.

First, we find that both sequencing and tiling analysis yielded very similar transcriptome maps, as had previously been found in *Schizosaccharomyces pombe *[14]. The transcribed regions identified corresponded extremely well between these two technologies, and the levels of expression of either genes or individual exons were very similar. Since the methodologies underlying tiling arrays and sequencing are very different, this suggests that both methods are providing accurate maps of the transcriptome and expression levels. Second, we used both methodologies to assess levels of alternative splicing in the worm, and to identify changes in splicing across development. We find that most of the alternative splicing events inferred from tiling arrays can be directly validated by sequence reads spanning exon junctions and find that at least 5% of genes have major changes to their isoforms between any two developmental stages. This suggests that using either technology, or a combination of both, to examine perturbations in alternative splicing either in different conditions or different mutant backgrounds will be very powerful. Finally, we used tiling arrays to identify over 10,000 regions of the *C. elegans *genome that are transcribed but lie outside current canonical gene models. We find that over 60% of these can be confirmed by sequence, suggesting that most of these are real transcripts. Other gene finding programs such as Twinscan predict many of these, indicating that there may be a systematic bias against these specific transcribed regions in the models used to build the current canonical gene models. We examined our sequence data and find that approximately 40% of these novel transcribed regions can be connected to current gene models; these are thus novel exons. The remainder likely represent entirely novel genes and their identification in this way may guide future refinements to both final gene models and *de novo *gene-finding algorithms.

Having made a 'reference' wild-type transcriptome, we used this to examine how this transcriptome is perturbed in worms lacking a functional NMD pathway. Transcripts containing PTCs are normally degraded in wild-type animals but will be retained in NMD mutants and such transcripts will thus be expressed at higher levels in the mutant. We find that in *C. elegans *(as in yeast, fly and human) approximately 10% of genes have higher expression levels in NMD mutant animals. First, there is a clear enrichment for the presence of an uORF; second, such transcripts are more likely to have a long 3' UTR; third, we see clear evidence for intron retention in many of these transcripts; and finally, we identify many transcripts that appear to be direct targets of NMD due to alternative splicing events. Taken together, we can identify one or more such features in over 55% of genes that have at least 1.5-fold increased expression in any single developmental stage in *smg-1(r861) *animals. This number rises to over 80% for genes that have higher expression in the *smg-1(r861) *mutant in all four stages. This suggests that most increases in gene expression seen in *smg-1(r861) *animals are direct effects of loss of NMD and that the genes whose expression is increased in *smg-1(r861) *mutant animals make PTC-containing transcripts that are normally degraded via NMD.

Identifying genes with perturbed expression in NMD mutants could have been done using conventional expression arrays (as was previously done in yeast, fly, and human). Since the resolution with which we can examine the transcriptome is far higher using tiling and sequencing, we also investigated whether other more subtle changes can be seen in the transcriptome of NMD mutant animals. We examined our data to determine whether we could detect any changes in splicing in any genes between the wild-type and mutant transcriptomes that would not have been possible using conventional expression arrays. We identified a large number of introns that have increased expression in NMD mutant animals. We infer that they are likely to be retained in both wild-type and NMD mutant animals due to inefficient splicing, causing PTCs - these PTC-containing transcripts are then degraded in wild-type animals but persist (and hence are present at higher levels) in the NMD mutants. Intriguingly, the retained introns do not appear to be a random set - fewer of these have the 'TTTCAG' splice site consensus at their 3' end and we thus suggest that efficiency of excision of introns flanked by splice sites that closely match the canonical sequences is far higher than for introns that have more divergent splice sites. In total, approximately 7% of genes give rise to transcripts with retained introns - these are usually degraded in wild-type animals. Overall, however, we find that intron excision is highly efficient - the vast majority of introns are undetectable in either wild-type or NMD mutant transcriptomes, and those that fail to be excised (and hence have elevated levels in NMD mutants) are usually present at low levels compared with overall gene expression levels.

Finally, we identified a set of genes that have different isoform levels in wild-type and NMD mutant transcriptomes. Intriguingly, the majority of genes that show a difference in splicing in the NMD mutants do not differ in overall expression level between the mutants and the wild-type animals. We infer from this that the difference in isoform levels in the mutant is not due to the retention of isoform variants that contain PTCs (as this would manifest itself in a detectable difference in expression levels) in the mutants but is instead an indirect consequence of a loss of NMD. In this case, rather than the pattern of splicing being identical in wild-type and mutant and differences in isoform levels being caused by a loss of NMD in the mutant, the pattern of splicing itself is different between wild-type and mutant. Previous data from a variety of organisms suggested that some splice factors themselves are targets of NMD and indeed we find that approximately 30% of all well-annotated splice factor genes appear to make transcripts that are NMD targets. More intriguing is the finding that genes encoding splicing factors are far more likely to produce PTC-containing splice variants than random sets of genes (30% of splice factors, 13% of other genes). This finding supports the view that alternative splicing and NMD are two highly interlinked processes [49,50,53,55-61].

We found that many of the splice changes seen in NMD mutant animals are likely to be indirect consequences of loss of NMD. Since many genes encoding splice factors are NMD targets, could this account for the indirect differences in splicing to many other genes seen in NMD mutant animals? We believe that this may be the case and that this may be the result of one of two possible effects. One alternative is that the level of splice factors is perturbed in animals defective for NMD and that this leads to the many changes in splicing seen in these mutants. Splice site selection is known to be highly sensitive to expression levels of splice factors and this is a plausible explanation. However, we note that although we detect a difference in overall transcript levels for many splice factors in NMD mutant animals, this does not imply that the levels of expressed full-length splice factor proteins differ in these animals compared with wild-type. The difference in expression levels of approximately 30% of splice factors in *smg-1(r861) *mutants is due to the synthesis of both full-length encoding transcripts that are stable in both wild-type and NMD mutant animals and PTC-including transcripts that are degraded in wild-type animals but not in animals lacking NMD. We suggest that the retention of these PTC-containing splice factor-encoding transcripts in *smg-1(r861) *animals may result in expression of truncated proteins that interfere with splice site specification by the full-length splice factors. The NMD pathway evolved to prevent precisely these kinds of detrimental effects following the expression of truncated proteins from PTC-containing transcripts. We note that this model is speculative and that the two alternatives are not mutually exclusive and our data alone cannot distinguish between these two models.

Intron retention, incorrect exon splicing, and the inheritance of nonsense mutations all lead to transcripts with PTCs and, thus, these are all classical targets of NMD. Even if such transcripts were translated, they cannot make full-length protein. The activation of NMD to degrade a transcript due to the presence of an uORF in the 5' UTR is more intriguing, however, since the principal ORF has no premature stops and thus could be translated. Current models suggest that the uORF stop is 'seen' by the NMD machinery as premature since the rest of the transcript is seen as an artificially elongated 'faux' 3' UTR [23,48,62-66] - our data are entirely consistent with this. However, we also find an intriguing correlation between the sequences surrounding the true ATG of the main ORF and the levels with which NMD recognizes uORF-containing transcripts for degradation. Even in transcripts containing an uORF, if the true ATG is surrounded by sequences that conform closely to a Kozak consensus, and thus is used efficiently for translational initiation, the transcript is less likely to be an NMD target. The effect is slight and poorly predictive - it does not explain why most transcripts containing an uORF evade NMD, and many other determinants must affect this. However, it fits well with a model where a first exploratory round of translation is used to find the start ATG followed by subsequent rounds of steady-state translation [67]. If the true ATG is in an excellent initiation consensus, it will be used in subsequent rounds of initiation and the uORF will be translated at a lower rate; hence, there will be a lower impact of NMD. The relationship between the efficiency of translational initiation at the true ATG and the level of NMD targeting suggests that, in transcripts containing a uORF, selection of sequences surrounding the true ATG may in some cases fine-tune protein levels not only by affecting the rates of translational initiation but also by affecting transcript turnover and hence transcript levels. It will be interesting to see whether this speculative model holds true as experimentalists studying NMD identify the exact sequence features that mark out targets of NMD.

Examining the transcriptome of NMD mutant animals has given us a window into the change in cellular transcription and transcript processing machinery since we can see all transcripts that are made in the wild-type animals but degraded by NMD. We find that, in general, transcription in the worm appears to be very specific - the great majority of transcripts identified in the NMD mutants are also present in wild-type animals and are not affected by loss of NMD. The cell thus does not appear to make a large quantity of aberrant PTC-containing transcripts. Splicing appears to be slightly more error-prone, however, with approximately 7% of genes making transcripts that are NMD targets due to a failure to excise introns. Finally, while some changes in splicing lead to PTCs in transcripts, many of the differences in splicing that we can detect in NMD mutants could be indirect, resulting from perturbations in transcripts encoding splice factors themselves. The high resolution transcriptome map that we used in this study was invaluable, allowing us not only to analyze expression levels of predicted genes, but also to examine each exon and intron individually as well as to identify novel transcribed regions. We hope that the availability of this scaffold will help direct future transcriptome research in *C. elegans*, in particular in the analysis of splicing and RNA stability.

## Materials and methods

### Strain maintenance and RNA preparation and processing

*C. elegans *strains were maintained on NGM agar plates seeded with OP50 *Escherichia coli *according to standard protocols [68]. Strains for which data are presented in this paper are Bristol N2, *smg-1(r861) *and *smg-5(r860)*. All strains were supplied by the *Caenorhabditis *Genetics Centre (CGC) [69], University of Minnesota, USA. Total RNA was prepared using Trizol solution according to the manufacturer's protocol, cleaned using Rneasy columns (QIAGEN, Venlo, Limburg, The Netherlands) and Dnase treated for 30 minutes with 10 U Dnase I (Roche, (Basel, Switzerland) in 1× One-Phor-All buffer (GE Healthcare, Little Chalfont, Buckinghamshire, UK). RNA was then re-purified using Rneasy columns before labeling and hybridizing to Affymetrix GeneChip *C. elegans *Tiling 1.0R Arrays as previously described [11,13]. In the case of sequence data, polyA+ RNA was purified from total RNA using Oligotex Midi Kits (QIAGEN) according to the manufacturer's protocol. cDNA was then produced using SuperScript™ Double-Stranded cDNA Synthesis Kit (Invitrogen, Carlsbad, CA, USA) and purified using a QIAGEN PCR Purification Kit. Sequence data for the resulting cDNA were then obtained as described by Wilhelm *et al. *[14].

### Processing of tiling microarray data

Raw spot intensity files (.CEL files) were quantile normalized in R. The normalized data were processed and exported as .BAR files using Affymetrix Tiling Analysis Software version 1.1 for visualization in Affymetrix Integrated Genome Browser. A background cutoff was calculated to include the top 5% of all non-genic probes (relative to WS150) for each condition and interval analysis then performed in Tiling Analysis Software to identify transcribed regions (transfrags) [11,13] above this cutoff. Maxgap and minrun parameters were set as 35 bp and 70 bp, respectively [11,13]. Genes were considered expressed if ≥50% of probes were above background in ≥50% of unique exons. Gene intensities of median exonic probes above background within filtered exons were then calculated. Exon intensities used for the splicing analysis were the median probe intensity of probes above background in the exons for which ≥50% of probes were above background.

### Normalized intensity and splice index calculation

NI was calculated for all internal exons (that is, cassette exons) as defined by WS150 gene annotations. NI for each exon was calculated as the ratio of expression of the exon relative to the expression of the gene:



The change in NI between any two stages, called the 'splice index' (SI), was calculated for each exon - this defines the change in expression of an exon relative to the expressed gene between conditions. More specifically:



where *E*_*i *_is the median probe intensity above background of the exon, *G*_*i *_of the gene and *t*_1 _and *t*_2 _are the different time points.

### Mapping sequence data to the genome

Reads obtained from sequencing were mapped to the genome using MAQ [43] version 0.6.6. A quality threshold of 30 was used as cutoff to determine aligned reads. This yields a count for the number of reads assigned to each nucleotide position in the genome. Each nucleotide can therefore be given an intensity score, which is the number of times it occurs in mapped reads. Gene intensities from sequence data are therefore calculated as the median number of reads that map to each nucleotide for which there is at least one read that corresponds to the given gene. Exon and intron expression are calculated as the median number of reads mapping across the exon or intron.

### Identifying reads mapping to splice sites

To map reads to splice junctions, we created a non-redundant set of sequences 66 nucleotides long corresponding to all possible splice junctions (annotated adjacent and non-adjacent exons based on WS150 were used; see Additional data file 6). This was created by combining 33 nucleotides from the 3' end of the upstream exon with 33 nucleotides from the 5' end of the downstream exon. Sequence reads were first mapped to the genome and the set of reads that mapped at less than a quality threshold of '30' by using MAQ were deemed unmapped. These reads were then aligned to the splice junctions, created as stated above, using BLAT and reads were identified as mapping to a junction if there was at least a four-nucleotide overlap over either exonic half of the corresponding junction. Reads that had multiple hits to different junctions were eliminated. Junction sequences formed by combining non-adjacent exons and having reads mapping to them uniquely were determined to be alternative splice sites. Currently, the calls are of a binary nature, with every alternative junction with reads mapping to it under the above criteria considered positive calls.

### Data access

The raw data can be accessed from two independent locations. The first is at Wormbase - all the tiling data and sequence data will be available as a download. The tiling data are viewable in Wormbase as tracks in the Gene View section. The sequence data are available from the NCBI Short Read Archive and the tracking number is SRA009279.

## Abbreviations

ENT: endogenous NMD target; MAQ: Mapping and Assembly with Qualities; NI: normalized intensity; NMD: nonsense-mediated decay; ORF: open reading frame; uORF: upstream ORF; PTC: premature termination codon; transfrag: transcribed feature; UTR: untranslated region.

## Authors' contributions

ACN, IB and PK performed the experiments. AKR and ACN analyzed the data. AGF, PK and TRG conceived of the study. All the authors participated in its design and coordination. AGF drafted the manuscript. All authors read and approved the final manuscript.

## Additional data files

The following additional data files are available with the online version of this paper: Tables S1 to S8 (Additional data file 1); a figure showing the distribution of mapped sequence reads (Additional data file 2); a figure detailing a comparison of tiling and sequence data for *smg-1(r861)*, which is analogous to Figure 1 for N2 (wild type) (Additional data file 3); a text file that contains the set of genes that are over-expressed twofold or more in the NMD mutant compared to wild type (Additional data file 4); a figure showing the structural changes in SR gene transcripts between N2 and *smg-1(r861) *(Additional data file 5); a fasta file containing all the exon junction sequences (Additional data file 6).

## Supplementary Material

Additional data file 1 Table S1 shows the number of genes identified as expressed at each stage in wild-type (N2) and *smg-1(r861) *worms. Table S2 shows the transfrag distribution at each developmental stage. Table S3 shows numbers of reads from sequencing and mapping statistics. Table S4 shows the number of tiling array transfrags confirmed by sequencing. Table S5 shows the overlap of genes detected between our data and that from Hillier *et al. *[17]. Table S6 shows the number of transfrags identified using tiling data, and the number of these also detected by either our sequence data or the Hillier *et al. *sequence data. Table S7 indicates the average ratio of expression for the families of splicing factors between the mutant and wild type at each of the time points based on both tiling and sequencing. Table S8 shows the list of 132 hand-curated splice factors. Click here for file

Additional data file 2 Distribution of mapped sequence reads. Click here for file

Additional data file 3 This figure is analogous to Figure 1 for N2 (wild type). Click here for file

Additional data file 4 For each gene the length of the 3' UTR (if greater than average), occurrence of uORFs and the presence of introns that are expressed in the mutant are listed. Click here for file

Additional data file 5 Structural changes in SR gene transcripts between N2 and *smg-1(r861)*. Click here for file

Additional data file 6 Each sequence was created by combining 33 nucleotides from the 3' end of the upstream exon with 33 nucleotides from the 5' end of the downstream exon using the WS150 release of the Wormbase gene models. Click here for file
