# Supplementary material for: High resolution transcriptome maps for wild-type and nonsense-mediated decay-defective Caenorhabditis elegans
Source: Genome Biol. 2009 Sep 24;10(9):R101. doi: 10.1186/gb-2009-10-9-r101 (PMC2768976; doi:10.1186/gb-2009-10-9-r101)
Supplement: Additional data file 1 — Table S1 shows the number of genes identified as expressed at each stage in wild-type (N2) and smg-1(r861) worms. Table S2 shows the transfrag distribution at each developmental stage. Table S3 shows numbers of reads from sequencing and mapping statistics. Table S4 shows the number of tiling array transfrags confirmed by sequencing. Table S5 shows the overlap of genes detected between our data and that from Hillier et al. [17]. Table S6 shows the number of transfrags identified using tiling data, and the number of these also detected by either our sequence data or the Hillier et al. sequence data. Table S7 indicates the average ratio of expression for the families of splicing factors between the mutant and wild type at each of the time points based on both tiling and sequencing. Table S8 shows the list of 132 hand-curated splice factors. [file gb-2009-10-9-r101-S1.DOC]

**Table S1**

|  | **Stage** | **Tiling** | **Sequence** | **Overlap (%)** |
| --- | --- | --- | --- | --- |
| **N2** | **L3** | 7208 |  |  |
|  | **L4** | 6355 | 8956 | 88 |
|  | **Young adults** | 7220 | 9120 | 90 |
|  | **Gravid adults** | 6577 |  |  |
| ***smg-1*** | **L3** | 7032 |  |  |
|  | **L4** | 6873 | 9235 | 88 |
|  | **Young adults** | 7526 | 10391 | 89 |
|  | **Gravid adults** | 7056 |  |  |

**Table S1. Number of genes detected by each technology and overlap.** The table shows the number of genes identified as expressed in each stage on wild-type (N2) and *smg-1(r861)* worms.

**Table S2**

|  | **Stage** | **Transfrags** | **Genic** | **Percent** | **Exonic** | **Percent** | **Novel** | **Percent** |
| --- | --- | --- | --- | --- | --- | --- | --- | --- |
| **N2** | **L3** | 49968 | 47717 | 95.50 | 45219 | 90.50 | 2251 | 4.50 |
|  | **L4** | 45770 | 43804 | 95.70 | 42050 | 91.87 | 1966 | 4.30 |
|  | **Young adults** | 46126 | 44139 | 95.69 | 42644 | 92.45 | 1987 | 4.31 |
|  | **Gravid adults** | 43507 | 41439 | 95.25 | 40045 | 92.04 | 2068 | 4.75 |
| ***smg-1*** | **L3** | 62993 | 58342 | 92.62 | 53516 | 84.96 | 4651 | 7.38 |
|  | **L4** | 55603 | 52367 | 94.18 | 49788 | 89.54 | 3236 | 5.82 |
|  | **Young adults** | 49465 | 47160 | 95.34 | 45514 | 92.01 | 2305 | 4.66 |
|  | **Gravid adults** | 50156 | 47265 | 94.24 | 45299 | 90.32 | 2891 | 5.76 |

**Table S2. Transfrag distribution at each developmental stage.** The great majority of transfrags detected are genic suggesting that the *C. elegans* genome is well annotated and there is little novel transcription.

**Table S3.**

|  | **Total Reads** | **Total Mapped** | **Percent** | **No of Lanes** |
| --- | --- | --- | --- | --- |
| **N2 Mixed** | 128,840,628 | 109,712,307 | 85.15 | 16 |
| **N2 L4** | 53,334,656 | 45,324,860 | 84.98 | 6 |
| **N2 YA** | 41,072,491 | 34,962,874 | 85.12 | 6 |
| ***smg-1* L4** | 32,949,897 | 26,661,967 | 80.92 | 5 |
| ***smg-1* YA** | 42,696,430 | 35,882,581 | 84.04 | 6 |

**Table S3. Number of reads from sequencing and mapping statistics.** Nearly 85% of sequence reads can be mapped uniquely to the genome at a MAQ mapping quality of ≥30.

**Table S4.**

|  | **Stage** | **Transfrags** | **% with sequence** | **Genic** | **% with sequence** | **Exonic** | **% with sequence** | **Novel** | **% with sequence** |
| --- | --- | --- | --- | --- | --- | --- | --- | --- | --- |
| **N2** | **L4** | 45770 | 92.86 | 43804 | 94.79 | 42050 | 96.38 | 1966 | 49.90 |
|  | **Young adult** | 46126 | 91.22 | 44139 | 92.83 | 42644 | 94.16 | 1987 | 55.36 |
| ***smg-1*** | **L4** | 55603 | 87.18 | 52367 | 89.66 | 49788 | 91.92 | 3236 | 47.06 |
|  | **Young adult** | 49465 | 93.78 | 47160 | 95.20 | 45514 | 96.40 | 2305 | 64.60 |

**Table S4. Tiling array transfrags confirmed by sequencing.** This table represents the proportions of genic, exonic and extra-genic transfrags validated by sequencing for the stages at which we have stage-specific sequence data. We note that more genic than extra-genic (novel) transfrags are validated by the sequence data. ~91-93% of all transfrags are validated at stages for which we have stage-specific sequence data.

**Table S5**

|  | **Ramani *et al*.**  **Tiling** | **Ramani *et al.***  **sequence** | **Hillier *et al.*** | **Overlap (Ramani *et al.* Tiling and Hillier *et al.)*** | **%** | **Overlap (Ramani *et al.* sequence and Hillier *et al.)*** | **%** |
| --- | --- | --- | --- | --- | --- | --- | --- |
| **L4** | 6355 | 8956 | 9004 | 5736 | 81.95 | 7506 | 73.25 |
| **YA** | 7223 | 9120 | 8619 | 6794 | 92.45 | 7992 | 84.11 |

**Table S5. Gene expression overlap between current analysis and Hillier *et al.*** We compare the set of genes identified as expressed in our tiling analysis with those identified from our sequence data and sequence data from Hillier *et al.* and find a very high overlap.

**Table S6**

|  | **L4** | | | | | **YA** | | | | |
| --- | --- | --- | --- | --- | --- | --- | --- | --- | --- | --- |
|  | **Total** | **Ramani *et. al*** | **Hillier *et al.*** | **Overlap** | **%** | **Total** | **Ramani *et. al*** | **Hillier *et al.*** | **Overlap** | **%** |
| **Exonic** | 42050 | 38990 | 38736 | 38210 | 98.00 | 42644 | 39460 | 39859 | 39106 | 99.10 |
| **Genic** | 1754 | 1092 | 1113 | 971 | 88.92 | 1495 | 939 | 996 | 879 | 93.61 |
| **Novel** | 1966 | 1083 | 1085 | 976 | 90.12 | 1987 | 1231 | 1278 | 1159 | 94.15 |

**Table S6. Comparion of transfrags identified using tiling data with sequence data.** The table shows a very high and consistent overlap between the transfrags identified from our tiling analysis with our sequence data and sequence data from Hillier *et al.*

**Table S7.**

|  | **L4.TilingRatio** | **L4.Seq.Ratio** | **YA.TilingRatio** | **YA.Seq.Ratio** |
| --- | --- | --- | --- | --- |
| **SR** | **1.67** | **1.93** | **1.44** | **1.11** |
| **hnRNP** | **1.54** | **1.87** | **1.34** | **1.62** |

**Table S7. Gene intensities of splice factor families.** The table indicates the average ratio of expression for the splice factors between the mutant and wild-type at each of the time points based on both tiling and sequencing. We see that the SR and the hnRNP families of splice factors are almost 1.5 fold upregulated in the mutants compared to wild-type.

**Table S8.**

| **Family** | **WBGeneID** | **Gene Public Name** |
| --- | --- | --- |
|
| snRNP | WBGene00014218 | *ZK1098.1* |
| snRNP | WBGene00016808 | *C50D2.5* |
| snRNP | WBGene00004187 | *prp-8* |
| snRNP | WBGene00004188 | *prp-21* |
| snRNP | WBGene00008683 | *F11A10.2* |
| snRNP | WBGene00011758 | *T13H5.4* |
| snRNP | WBGene00011605 | *T08A11.2* |
| snRNP | WBGene00021004 | *W03F9.10* |
| snRNP | WBGene00019323 | *tag-203* |
| snRNP | WBGene00004723 | *sap-49* |
| snRNP | WBGene00017605 | *F19F10.9* |
| snRNP | WBGene00010896 | *M28.5* |
| snRNP | WBGene00017280 | *F09D1.1* |
| snRNP | WBGene00004390 | *rnp-7* |
| snRNP | WBGene00004385 | *rnp-2* |
| snRNP | WBGene00004386 | *rnp-3* |
| snRNP | WBGene00017238 | *F08B4.7* |
| snRNP | WBGene00044076 | *sap-1* |
| snRNP | WBGene00007972 | *prp-4* |
| snRNP | WBGene00010844 | *M03C11.7* |
| snRNP | WBGene00017162 | *ddx-23* |
| snRNP | WBGene00013343 | *Y59A8B.6* |
| snRNP | WBGene00001166 | *eft-1* |
| snRNP | WBGene00012896 | *Y46G5A.4* |
| snRNP | WBGene00015974 | *C18E3.5* |
| snRNP | WBGene00008586 | *F08G12.2* |
| U2AF | WBGene00006698 | *uaf-2* |
| U2AF | WBGene00006697 | *uaf-1* |
| Sm | WBGene00003076 | *lsm-1* |
| Sm | WBGene00001808 | *gut-2* |
| Sm | WBGene00003077 | *lsm-3* |
| Sm | WBGene00003078 | *lsm-4* |
| Sm | WBGene00003079 | *lsm-5* |
| Sm | WBGene00003080 | *lsm-6* |
| Sm | WBGene00003081 | *lsm-7* |
| Sm | WBGene00003082 | *lsm-8* |
| Sm | WBGene00004915 | *snr-2* |
| Sm | WBGene00004916 | *snr-3* |
| Sm | WBGene00004917 | *snr-4* |
| Sm | WBGene00004914 | *snr-1* |
| Sm | WBGene00004919 | *snr-6* |
| Sm | WBGene00004918 | *snr-5* |
| Sm | WBGene00004920 | *snr-7* |
| SR | WBGene00004703 | *rsp-6* |
| SR | WBGene00013293 | *Y57G11A.5* |
| SR | WBGene00004704 | *rsp-7* |
| SR | WBGene00011035 | *R05D11.7* |
| SR | WBGene00004701 | *rsp-4* |
| SR | WBGene00013260 | *rsr-2* |
| SR | WBGene00004700 | *rsp-3* |
| SR | WBGene00004698 | *rsp-1* |
| SR | WBGene00004699 | *rsp-2* |
| SR | WBGene00004702 | *rsp-5* |
| SR | WBGene00004705 | *rsp-8* |
| hnRNP | WBGene00019249 | *H28G03.1* |
| hnRNP | WBGene00001999 | *hrp-1* |
| hnRNP | WBGene00022235 | *sqd-1* |
| hnRNP | WBGene00003978 | *pes-4* |
| hnRNP | WBGene00022253 | *Y73B6BL.33* |
| hnRNP | WBGene00020936 | *hrpf-1* |
| hnRNP | WBGene00004207 | *ptb-1* |
| hnRNP | WBGene00017816 | *F26B1.2* |
| hnRNP | WBGene00016624 | *C44B7.2* |
| hnRNP | WBGene00007706 | *C25A1.4* |
| hnRNP | WBGene00002000 | *hrp-2* |
| hnRNP | WBGene00012769 | *Y41E3.11* |
| hnRNP | WBGene00003423 | *msi-1* |
| RRM_Domain | WBGene00000770 | *cpb-1* |
| RRM_Domain | WBGene00000772 | *cpb-3* |
| RRM_Domain | WBGene00000774 | *cpf-2* |
| RRM_Domain | WBGene00000889 | *cyn-13* |
| RRM_Domain | WBGene00000890 | *sig-7* |
| RRM_Domain | WBGene00000935 | *daz-1* |
| RRM_Domain | WBGene00001230 | *eif-3.G* |
| RRM_Domain | WBGene00001340 | *etr-1* |
| RRM_Domain | WBGene00001481 | *fog-1* |
| RRM_Domain | WBGene00003827 | *ntl-4* |
| RRM_Domain | WBGene00003902 | *pab-1* |
| RRM_Domain | WBGene00003903 | *pab-2* |
| RRM_Domain | WBGene00003904 | *pabp-2* |
| RRM_Domain | WBGene00004315 | *rbd-1* |
| RRM_Domain | WBGene00004384 | *rnp-1* |
| RRM_Domain | WBGene00004387 | *rnp-4* |
| RRM_Domain | WBGene00004388 | *rnp-5* |
| RRM_Domain | WBGene00004389 | *rnp-6* |
| RRM_Domain | WBGene00004782 | *set-2* |
| RRM_Domain | WBGene00004984 | *spn-4* |
| RRM_Domain | WBGene00006321 | *sup-12* |
| RRM_Domain | WBGene00006367 | *sym-2* |
| RRM_Domain | WBGene00006514 | *tag-169* |
| RRM_Domain | WBGene00007111 | *B0035.12* |
| RRM_Domain | WBGene00007396 | *C07A4.1* |
| RRM_Domain | WBGene00008224 | *C50B8.1* |
| RRM_Domain | WBGene00008549 | *din-1* |
| RRM_Domain | WBGene00008688 | *F11A10.7* |
| RRM_Domain | WBGene00009141 | *ncbp-2* |
| RRM_Domain | WBGene00009314 | *F32B4.4* |
| RRM_Domain | WBGene00010677 | *K08F4.2* |
| RRM_Domain | WBGene00011043 | *R05H10.2* |
| RRM_Domain | WBGene00011059 | *R06C1.4* |
| RRM_Domain | WBGene00011155 | *R09B3.2* |
| RRM_Domain | WBGene00011156 | *R09B3.3* |
| RRM_Domain | WBGene00011199 | *tag-310* |
| RRM_Domain | WBGene00011279 | *asd-1* |
| RRM_Domain | WBGene00011408 | *T04A8.6* |
| RRM_Domain | WBGene00011589 | *T07F10.3* |
| RRM_Domain | WBGene00011722 | *T11G6.8* |
| RRM_Domain | WBGene00011730 | *drr-2* |
| RRM_Domain | WBGene00012245 | *W04D2.6* |
| RRM_Domain | WBGene00012558 | *Y37D8A.21* |
| RRM_Domain | WBGene00013307 | *Y57G11C.9* |
| RRM_Domain | WBGene00013703 | *Y106G6D.7* |
| RRM_Domain | WBGene00015329 | *C02B10.4* |
| RRM_Domain | WBGene00015581 | *C07H6.4* |
| RRM_Domain | WBGene00016173 | *C27H5.3* |
| RRM_Domain | WBGene00016245 | *C30B5.4* |
| RRM_Domain | WBGene00016653 | *C44E4.4* |
| RRM_Domain | WBGene00017004 | *nrd-1* |
| RRM_Domain | WBGene00017138 | *EEED8.10* |
| RRM_Domain | WBGene00017140 | *EEED8.12* |
| RRM_Domain | WBGene00017929 | *F29C4.7* |
| RRM_Domain | WBGene00019241 | *H24K24.4* |
| RRM_Domain | WBGene00019510 | *K07H8.10* |
| RRM_Domain | WBGene00019881 | *R05D3.8* |
| RRM_Domain | WBGene00020091 | *rnp-8* |
| RRM_Domain | WBGene00020354 | *T08B6.5* |
| RRM_Domain | WBGene00020399 | *ztf-4* |
| RRM_Domain | WBGene00021901 | *tag-262* |
| RRM_Domain | WBGene00021921 | *Y55F3AM.3* |
| RRM_Domain | WBGene00021994 | *Y59E9AL.4* |
| RRM_Domain | WBGene00022025 | *Y65B4A.1* |
| RRM_Domain | WBGene00022771 | *ZK616.1* |

**Table S8. List of 132 hand-curated splice factors.** Genes annotated as splice factors or annotated to contain a RRM domain were hand-curated from Wormbase, pfam and SwissProt. The list contains proteins from the spliceosome, SM, SR, hnRNP families and also all proteins that conatin a RRM domain.
