# Supplementary material for: High resolution transcriptome maps for wild-type and nonsense-mediated decay-defective Caenorhabditis elegans
Source: Genome Biol. 2009 Sep 24;10(9):R101. doi: 10.1186/gb-2009-10-9-r101 (PMC2768976; doi:10.1186/gb-2009-10-9-r101)
Supplement: Additional data file 2 — Distribution of mapped sequence reads. [file gb-2009-10-9-r101-S2.PDF]

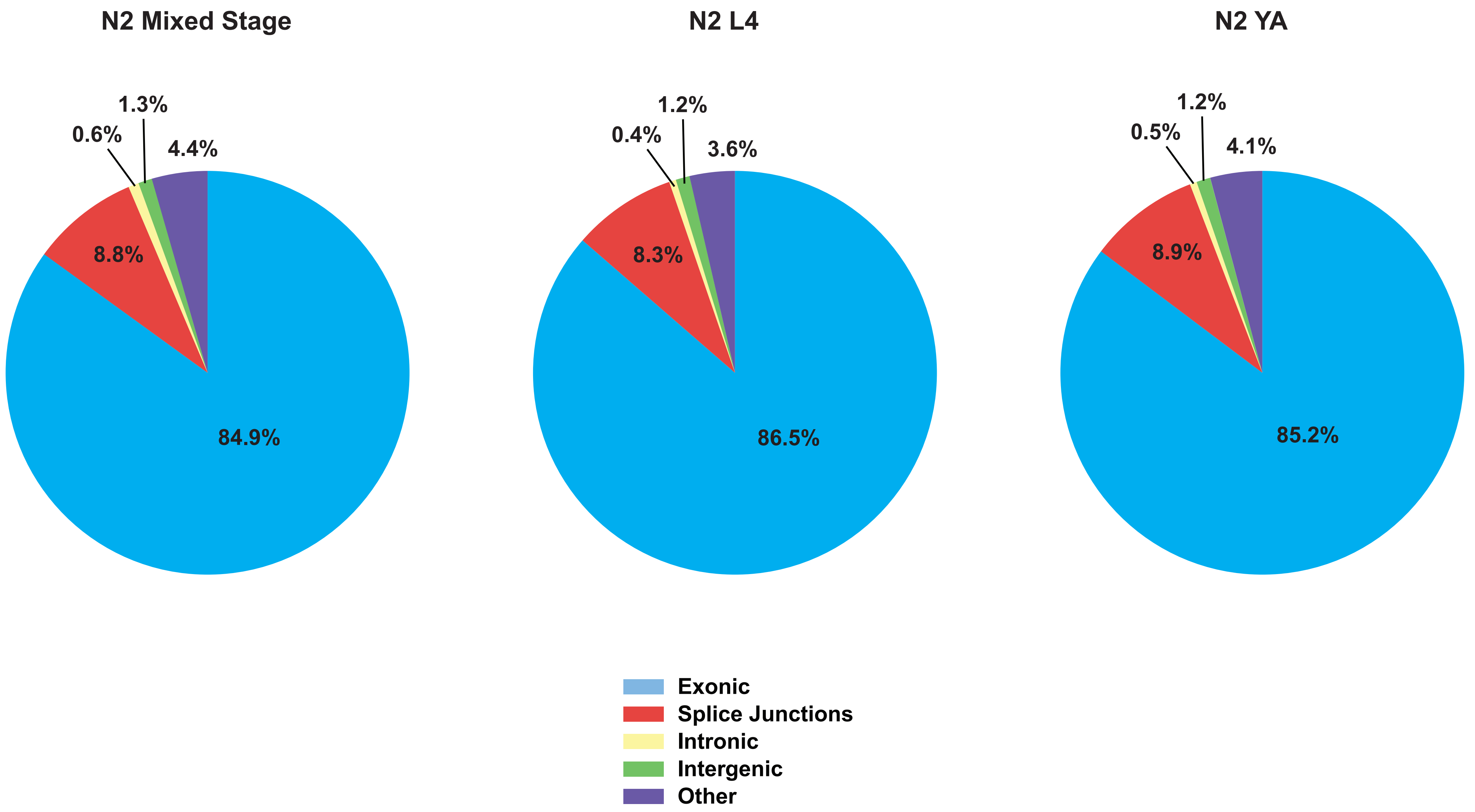

Figure S1. Distribution of mapped sequence reads. Approximately 85% of the reads maps to known exonic regions while 9% of the reads map to exon junctions, indicating a high level of correlation in read mapping to existing gene annotations. Less than 5% of the sequenced reads map to novel regions in the genome.
