# Supplementary material for: High resolution transcriptome maps for wild-type and nonsense-mediated decay-defective Caenorhabditis elegans
Source: Genome Biol. 2009 Sep 24;10(9):R101. doi: 10.1186/gb-2009-10-9-r101 (PMC2768976; doi:10.1186/gb-2009-10-9-r101)
Supplement: Additional data file 3 — This figure is analogous to Figure 1 for N2 (wild type). [file gb-2009-10-9-r101-S3.PDF]

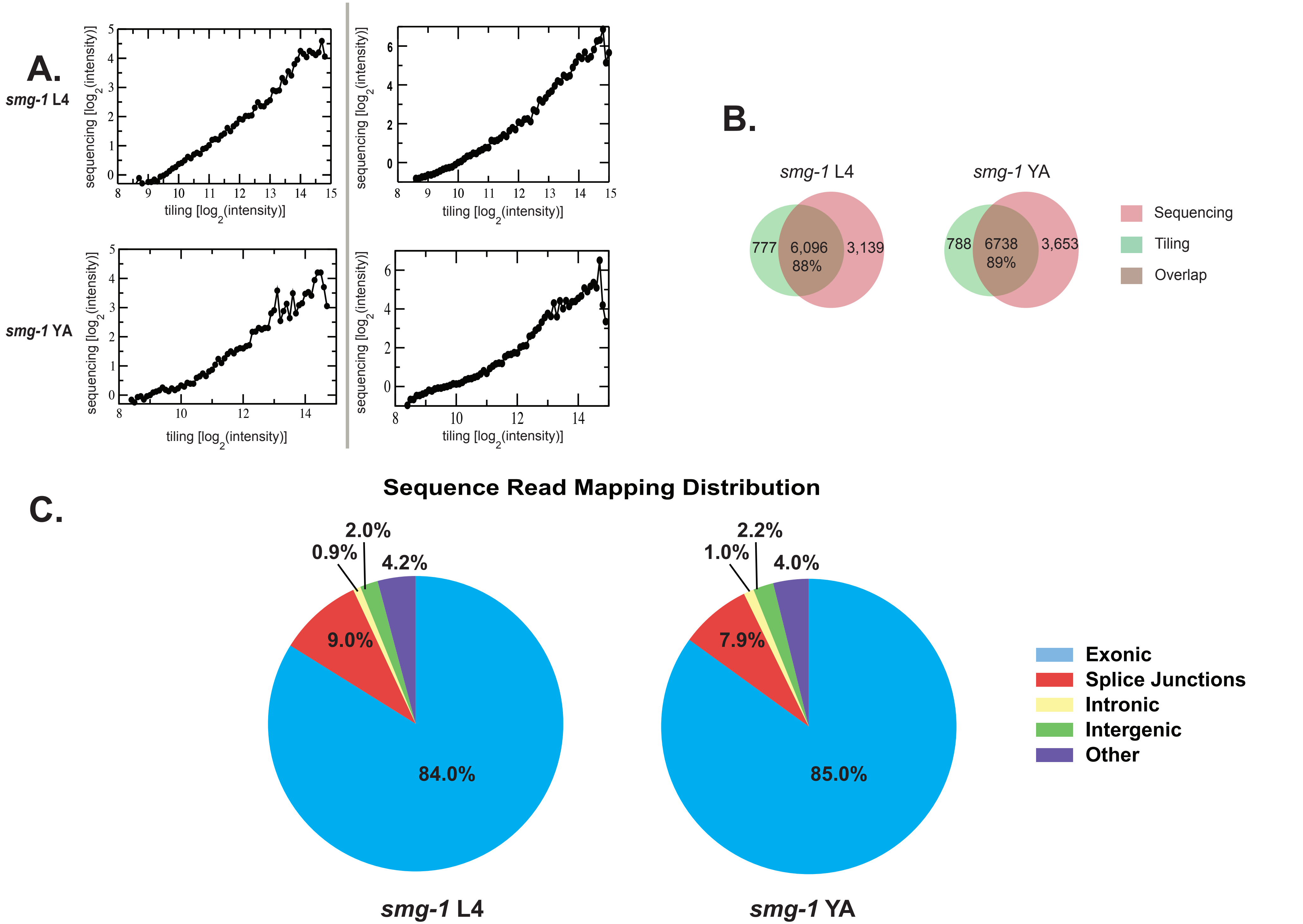

Figure S2. Comparison of tiling and sequence data for *smg-1(r861)* mutant. (a) correlation of gene intensities (left) and exon intensities (right) at two different developmental shows very high positive correlation. (b) Nearly 90% of genes expressed by tiling arrays are found to be expressed by sequencing as well. (c) As seen for the wild-type N2 sequence reads, nearly 95% of the sequencing reads from the mutants map to known transcripts.
