# Supplementary material for: High resolution transcriptome maps for wild-type and nonsense-mediated decay-defective Caenorhabditis elegans
Source: Genome Biol. 2009 Sep 24;10(9):R101. doi: 10.1186/gb-2009-10-9-r101 (PMC2768976; doi:10.1186/gb-2009-10-9-r101)
Supplement: Additional data file 5 — Structural changes in SR gene transcripts between N2 and smg-1(r861). [file gb-2009-10-9-r101-S5.PDF]

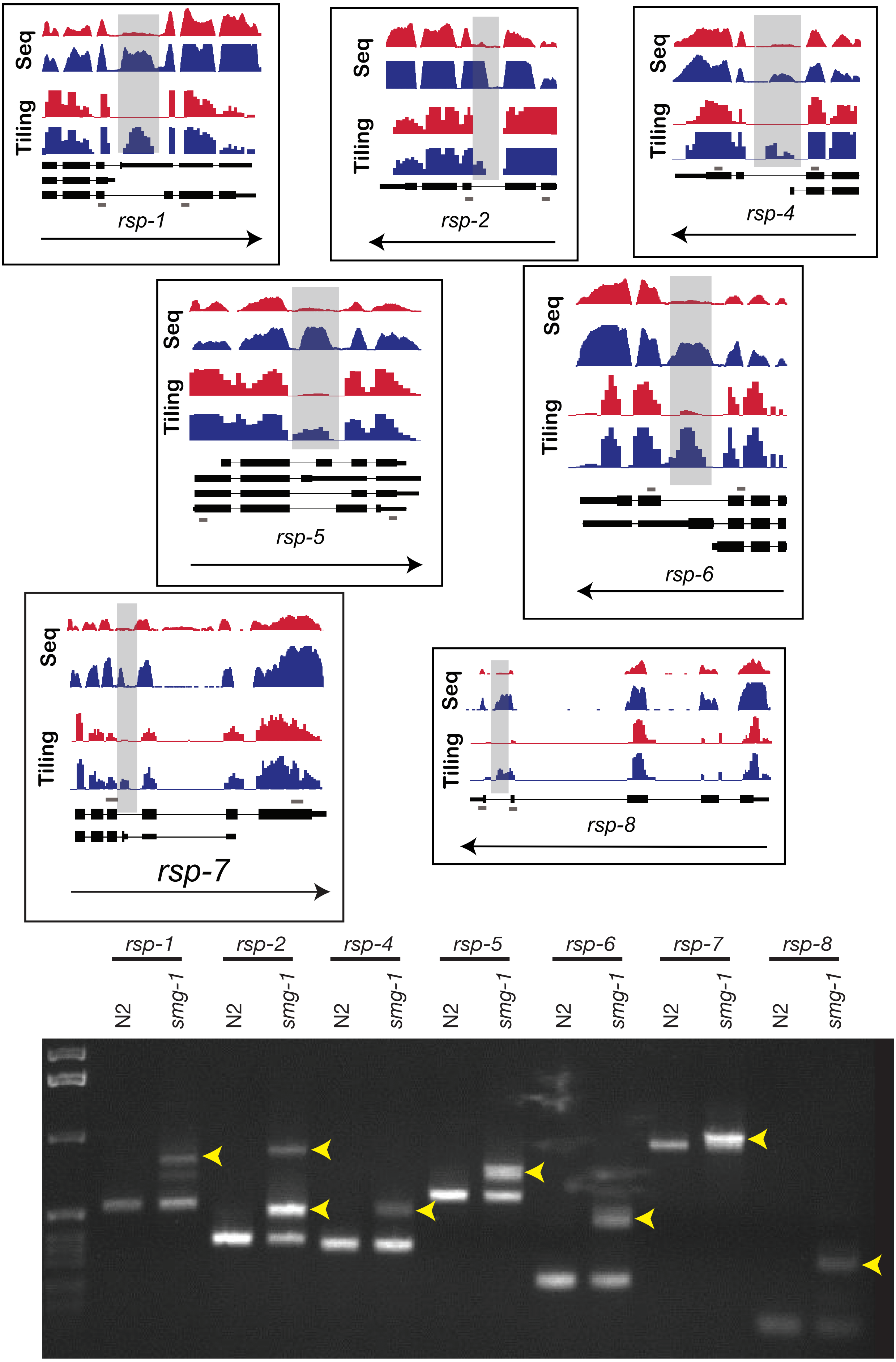

Figure S3. Structural changes in SR gene transcripts leading to NMD. Normalized probe intensities derived from N2 L4 stage animals is in blue and smg-1(r861) L4 animals in red. The top lanes correspond to the sequence data while the bottom lanes correspond to tiling data. The visually identified structural difference between the N2 and smg-1(r861) transcript(s) is indicated by the grey box. RT-PCR to amplify across this region was performed between flanking exons and the PCR products run on a gel. The positions of the primers used for RT-PCR are indicated with short grey bars. As can be seen in the gel image, a single band was detected for each gene in N2 but at least one additional larger product was seen in smg-1(r861). This suggests that NMD-targeted isoforms of these genes are produced. The largest band correlates with the inclusion of the full novel structure but intermediate bands imply that multiple splice events occur within.
